# Mir-21 and Mir-125b as theranostic biomarkers for epithelial ovarian cancer in Tunisian women

**DOI:** 10.4314/ahs.v23i2.29

**Published:** 2023-06

**Authors:** A Habel, F Nassar, M Itani, H Bouaziz, M Hadj-Ahmed, Z Msheik, M Stayoussef, R Nasr, B Yacoubi-Loueslati

**Affiliations:** 1 Laboratory of Mycology, Pathologies and Biomarkers (LR16 ES05), Faculty of Sciences of Tunis, University Tunis El Manar, Tunis, Tunisia; 2 Department of Internal Medicine, Naef K. basile Cancer Institute American University of Beirut, Beirut, Lebanon; 3 Department of Anatomy, Cell Biology and Physiological Sciences, American University of Beirut, Beirut, Lebanon; 4 Department of Carcinological Surgery, Salah Azaiez Institute, Tunis

**Keywords:** Epithelial ovarian cancer, miRNAs, biomarkers, resistance to treatment, Tunisia

## Abstract

**Background:**

Ovarian cancer (OC) is the third most common cancer in women and the leading cause of death associated with gynecologic tumors. Because this disease is asymptomatic in the early stages, most patients are not diagnosed until the late stages. This highlights the need for the development of diagnostic biomarkers. MicroRNAs (miRNAs), small non-coding RNAs, are currently being explored as potential biomarkers for the early detection of various malignancies in humans. However, their expression and diagnostic value in OC have not been well studied.

**Materials and Methods:**

the plasma levels of miR-21, miR-200a, miR-200b, miR-200c, miR-205 and miR-125b were determined in epithelial ovarian cancer (EOC) patients and healthy controls by Reverse Transcription Quantitative Realtime Polymerase Chain Reaction *(RT-qPCR)*. The expression levels of the deregulated microRNAs were analysed according to clinical characteristics.

**Results:**

It was found that miR-21 and miR-125b were upregulated in EOC compared with healthy controls. Moreover, decreased miR-125b was associated with resistance to platinum-based chemotherapy.

**Conclusions:**

Our data suggest that miR-21 and miR-125b in plasma may serve as potential circulating biomarkers for the early detection of EOC. MiR-125b may also be useful for predicting chemosensitivity in EOC patients.

## Introduction

Epithelial ovarian cancer (EOC) is the major form of ovarian cancer (OC) that severely threatens women's health worldwide. It has the highest mortality rate among all gynecologic cancers 1, 2. This is because it is not easily diagnosed at an early stage. EOC is often referred to as a ‘silent killer’ because it is a tumour that spreads in the abdomen and metastasizes without specific symptoms. Therefore, most EOC patients are not diagnosed until an advanced stage 2. Recent reports indicate that 80% of patients with advanced EOC have an inferior prognosis and relapse 3. To date, clinical treatment of EOC has been based on cytoreductive tumour surgery and a combination of paclitaxel and platinum-based chemotherapy. Unfortunately, EOC has both a high recurrence rate and drug resistance which causes a high mortality rate 4,5. Hence, to improve the survival rate of EOC patients, there is an urgent need to identify new diagnostic and theranostic biomarkers characterized by higher specificity and sensitivity.

Recently, microRNAs (miRNAs) have been analysed as potential biomarkers for the early detection of various types of cancer, including EOC 6,7 miRNAs were discovered as a new class of evolutionarily conserved small non-coding molecules 8. They play an important regulatory role in cellular homeostasis 9. They also control normal cell development, differentiation, and apoptosis and determine cancer cells' final phenotype 10. Several miRNAs are dysregulated in EOC 7. Some studies have reported that some miRNAs such as miR-15, miR-31, miR-155, miR-127, and miR-99b are downregulated in EOC compared with controls, suggesting that they function as tumour suppressors 11-13. Other miRNAs such as miR-199a, miR-200a, miR-200b, miR-200c, miR-21 and miR-125b are overexpressed in EOC and may be considered cancer promoters or oncomiRs 14,15. However, few studies have investigated the potential value of miRs as theranostic biomarkers for EOC.

In this regard, we analysed a panel of miRNAs: miR-21, miR-200a, miR-200b, miR-200c, miR-205, and miR-125b to evaluate their potential diagnostic and theranostic value in EOC.

## Materials and Methods

### Study subjects

Institutional review board (IRB) approval for this study was first obtained from the Salah Azaeiz Institute of Tunis (SAI) Research and Ethics committee (IRB reference: 01/ISA/2019). A total of 49 EOC patients (mean age = 52.47 ±12.72). All were newly diagnosed outpatients at the SAI surgical and oncology service between June 2019 and November 2020. All patients received no therapy at the time of recruitment and were followed for at least 6 months after treatment using their medical records. This was done to determine whether or not they responded to treatment or had a relapse.

Twenty-five age-matched healthy women who had no personal or family history of EOC or any type of cancer were included as healthy control subjects. None of the subjects considered (cases and controls) had any unrelated comorbidity. The clinicopathological characteristics of the patients were obtained from medical records and interviews using a structured questionnaire. Patients' medical records were also reviewed 6 months after sampling to monitor response to chemotherapy (CT). All patients and controls provided written informed consent.

### Blood sampling

Five millilitres of venous blood were collected in a sterile tube containing EDTA. Plasma samples were separated within 2 hours of collection using a ficoll gradient centrifuging at 2.500 rpm for 20 minutes. Next, plasma samples were transferred to a clean microcentrifuge tube and centrifuged at 14.000 rpm for 10 minutes to remove cell debris and fragments. Finally, plasma samples were aliquoted and stored.

### RNA extraction

#### RNA extraction and cDNA synthesis

Total RNA (including miRNA) was isolated from 250µl of plasma using the Plasma/Serum Circulating and Exosomal RNA Purification Kit (Norgen Biotek Corp., Thorold, ON, Canada) according to the manufacturer's instructions. The quantity and quality of the extracted RNA were determined using the DeNovix DS-11 FX spectrophotometer (Wilmington, DE, USA). For cDNA synthesis, reverse transcription of ten nanograms of the total RNA was performed using the TaqMan® MicroRNA Reverse Transcription Kit (Applied Biosystems, USA) according to the manufacturer's instructions 16,17. The reaction mixture was incubated at 16°C for 30 minutes, 42°C for 30 minutes, and 85°C for 5 minutes. Finally, the cDNA samples were diluted in 57µl DEPC water.

#### Reverse Transcription Quantitative Real-Time Polymerase Chain Reaction (RT-qPCR)

RT-qPCR for miR-21, miR-200a, miR-200b, miR-200c, miR-205, and miR-125b was performed using validated primers and probes from the TaqMan® microRNA Assays Kit, and 2x TaqMan® Universal Master Mix without Amperase Uracil N-glycosylase (UNG) (Applied Biosystems, Waltham, MA, USA). The reaction mix was incubated for 10 minutes at 95°C and 40 cycles of 10 seconds at 95°C and 60 seconds at 60°C using Bio-Rad CFX96 Real-Time System, C1000 Thermal Cycler (Hercules, CA, USA). The RT-qPCR reaction was performed in duplicate for miR-21, miR-200a, miR-200b, miR-200c, miR-205 and miR-125b. MiR-16 was used as an endogenous control. We calculated relative miR expression (fold change expression) using the equation 2^-ΔΔCt: ΔΔCt=ΔCt (EOC samples)-ΔCt (control samples) and ΔCt = Cq target (miR of interest)- Cq (miR-16) 18.

### Statistical analysis

Statistical analysis was performed using GraphPad Prism 7 and a p-value < 0.05 was considered statistically significant. Data are presented as mean ±SD. Differences between groups (EOC patients and controls) were compared using the Fold Change and Wilcoxon's Signed Rank test. Differences between subgroups of patients were determined using the Mann-Whitney- test or the Kruskal-Wallis's test.

## Results

### Study subject

Clinical and pathological data of 49 Tunisian EOC patients are summarized in table 1. Their mean age was (52.47±12.72). The mean body mass index (BMI) is (27.66±4.484) and 22.4% of patients were obese. According to the International Federation of Gynecology and Obstetrics (FIGO), 14 (28.5 %) patients were diagnosed with early-stage EOC (stage I and stage II), 35 (71.5%) patients were diagnosed with late-stage EOC (stage III and stage IV), and 19 (38.78%) patients had distant metastasis. All patients were treated with six doses of a combination of Taxol and carboplatin CT that can be used as an adjuvant (before surgery) or after surgery. This depends on the size, grade, and type of tumour. Nine (18.36%) cases were sensitive to chemotherapy (Taxol-Carbo) and fourteen recurred after six months of treatment (Table 1).

**Table 1:** Characteristics of study participants

		Cases n=49 (%)	Controls n=25 (%)	P
Age (years)^1^ (mean ±SD)		52.47±12.72	52.14±13.36	0.3914
BMI (kg/m^2^)^1^ (means ±SD)		27.66±4.484		0.5602
Menarche (years) (mean ±SD)		12.49±1.218	-	-
Nulliparous^2^	nulliparous	18(36.73)	-	-
	Uniparous	8 (16.32)		
	Multiparous	23 (44.93)		
Menopausal status^1^	Pre-menopausal	18 (32.8)	-	-
	Post-menopausal	31 (67.2)		
Users of oral contraception^1^	No	40 (81.6)	-	-
	Yes	9 (18.4)		
FIGO staging^1^	Early stage (I+II)	14 (28.5)	NA	NA
	Late stage (III + IV)	32 (71.5)		
Grade^1^	Low grade (I+ II)	28 (57.14)	NA	NA
	High grade (III)	21 (21.86)		
Recurrence of disease^1^	No	30 (61.22)	NA	NA
	Yes	19 (38.78)		
Distant metastasis ^1^	No	30 (61.22)	NA	NA
	Yes	19 (38.78)		
CA-125	<35 (U/ml)	10 (20.4))	-	-
	>35 (U/ml)	30 (61.2)		
	ND	9 (18.4)		
Response to chemotherapy^1^	Sensitive	40 (81.64)	NA	NA
	Resistant	9 (18.36)		

SD: standard deviation; BMI: Body Mass Index; FIGO International Federation of Gynaecology and Obstetrics

1 p value performed with Mann-Whitney test

2 performed with Kruskal-Wallis test

NA: Not applicable; ND: not determined

### Expression levels of miR-21 and miR-125b in plasma EOC patients and controls

**Figure 1:**
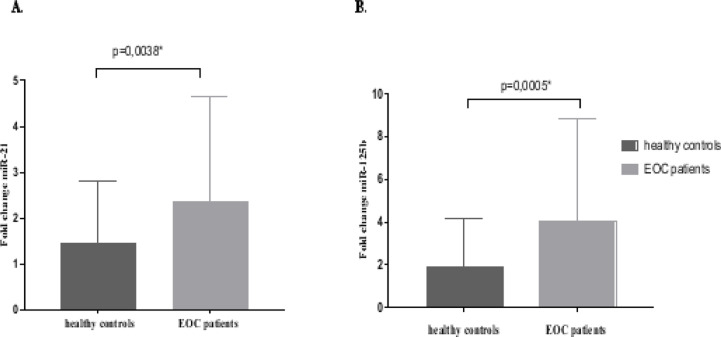
Upregulated miRNA expression in Tunisian Ovarian cancer plasma

The expression levels of miR-21, miR-200a, miR-200b, miR-200c, miR-205, and miR-125b were measured in the plasma of 49 newly diagnosed EOC patients and compared with their expression in the plasma of 25 healthy individuals. The expression levels of miR-21 and miR-125b in plasma were significantly higher in EOC patients compared to healthy controls (p=0.0038*; p=0.0005*, respectively) (figure 1). On the other hand, miR-200a, miR-200b, miR-200c, and miR-205 were not significantly dysregulated in the plasma of EOC patients (data not shown).

### Association between miR-21 and miR-125b and the pathological features of EOC

To better assess the possible association of miR-21 and miR-125b with the development of EOC, fold changes of these two miRs were compared between EOC patients stratified according to different clinicopathological characteristics such as age, BMI, nulliparity, menarche, menopause, oral contraception, FIGO staging, grade, disease recurrence, distant metastasis, and response to treatment. The results did not reveal any significant association between age, BMI, menarche, menopause, FIGO staging, grade, disease recurrence, distant metastasis and miRs plasma levels (Table 2). However, we observed that the expression of miR-21 significantly increased in multiparous women (p= 0.0439*) and women who took birth control pills (p= 0.0315*). In addition, the level of miR-125b was significantly higher in EOC patients who responded to chemotherapy (carbo-Taxol) than in those who developed resistance to this CT regimen (7.241 ±7.117 vs. 2.761 ±3.5; p=0.0457*) (Table 2).

**Table 2:** Comparison between relative expression of miR-21 and miR-125b and clinicopathological parameters

		miR-21		miR-125b	

		Fold change (means±SD)	p	Fold change (means±SD)	p
Age (years)^1^	≤52	3.889±3.849	0.4887	5.59±6.082	0.9507
	>52	3.455±3.81		3.72±7.197	
BMI (kg/m^2^)^1^	Underweight + normal	2.635±3.044	0.1040	3.358±4.566	0.1267
	Overweight + obese	4.402±4.085		6.755±7.033	
Menarche (years)^2^	>12	3.766 ±3.654	0.9563	1.267 ±1.227	0.051
	12-13	3.483 ±3.68		5.551 ±6.343	
	<14	3.93±4.4808		8.253±7.702	
Nulliparitous	nulliparous	3.059±3.907	0.00439*	3.796±4.964	0.1643
	Uniparous	1.27±1.15		6.929±9.609	
	Multiparous	4.81±3.897		6.455±6.314	
Menopausal status^1^	Pre-menopausal	2.281±2.49	0.1240	3.165±3.707	0.1251
	Post-menopausal	4.473±4.211		7.21 ±7.496	
Users of oral contraception ^1^	No	3.276±3.582	0.0315*	3.276 ±3.582	0.1730
	Yes	16.832±4.403		6.944 ±8.972	
FIGO staging^1^	Early stage (I+II)	2.833±2.863	0.5833	3.983 ±5.208	0.2873
	Late stage (III + IV)	3.989 ±4.094		6.396 ±7.041	
Grade^1^	Low grade (I+II)	3.776±3.825	0.6771	6.758 ±7.811	0.6519
	High grade (III)	3.847±3.906		4.632 ±4.405	
Recurrence of the disease^1^	No	3.85±3.84	0.7256	5.762 ±5466	0.3181
	Yes	3.373±3.809		5.498 ±8.072	
Distant metastasis ^1^	No	3.01±2.91	0.5214	5.613 ±6.244	0.9947
	Yes	4.678 ±4.777		5.713 ±7.202	
CA-125	<35 (U/ml)	4.263±3.947	0.9646	7.17 ±7.271	0.8491
	>35 (U/ml)	4.317±4.456		6.252 ±5.93	
Response to chemotherapy ^1^	Sensitive	4.1±3.86	0.4993	7.241±7.117	0.0457*
	Resistant	3.089±4.421		2.761±3.5	

BMI: Body Mass Index; SD: standard deviation; FIGO International Federation of Gynaecology and Obstetrics;

1 p value performed with Mann-Whitney test

2 p value performed with Kruskal-Wallis test

* p<0.05

## Discussion

EOC is a common gynecologic disease with an insidious onset that makes early detection difficult and is responsible for a high mortality rate (https://gco.iarc.fr/). Elucidating factors associated with higher risk and development and/or response to chemotherapy of EOC is essential to improve diagnosis and prognosis. miRNAs have been shown to influence numerous cellular processes such as proliferation, differentiation, and apoptosis. Their aberrant expression has been well document in various diseases, including cancer 19. Fortunately, miRNAs are stable and easily detected in plasma, so they have potential value as diagnostic biomarkers for multiple cancers, including EOC 20. In this regard, the association of dysregulated plasma levels of miR-21, miR-200a, miR-200b, miR-200c, miR-205, and miR-125b, and the risk of EOC was documented in several populations 21-24. In this study, we also confirmed the upregulation of circulating miR-21 and miR-125b in EOC patients compared to healthy controls.

Located on chromosome 17, miR-21 is one of the earliest identified cancer-promoting “oncomiR” targeting numerous tumour suppressor genes associated with proliferation, apoptosis and invasion 25. Our result shows that the expression of miR-21 was twice as high in EOC patients compared to healthy subjects (p<0.05). This is consistent with Chinese 23,27 , Egyptian 28, American 23, and Indian studies 29, which also documented increased levels of miR-21 in ovarian cancer patients. Furthermore, other studies have also demonstrated the overexpression of miR-21 in many malignancies such as breast cancer 30, head, and neck cancer 31,32, Hodgkin lymphoma 33, chronic myeloid leukemia 34, colon cancer 35, prostate cancer 36, brain tumour 37, cholangiocarcinoma 38, lung cancer 39, esophageal cancer 40 and pancreatic cancer 41 supporting the potential diagnostic and prognostic value of miR-21.

The increased levels of miR-21 can be explained by its close association with carcinogenesis, as it has been shown to interfere with cell survival by regulating the cell cycle, apoptotic proteins, metalloproteinases, and others 42. Thus, miR-21 is a potential key factor in tumour growth and the initiation, progression, invasion, and metastasis in various tumours, including EOC.

MiR-125b is ubiquitously expressed with the highest expression in the ovaries and brain (http://www.microrna.org/). miR-125b is dysregulated in a variety of tumours. Some studies have reported upregulation of miR-125b in some tumours such as colorectal cancer 43, gastric cancer 44, follicular carcinoma 45, and pancreatic cancer 46, suggesting an oncogenic potential of miR-125b. However, others have documented its downregulation in head and neck tumours 47, oral squamous cell carcinoma 48, osteosarcoma 49, bladder cancer 50,51, and thyroid cancer 45, highlighting its tumour-suppressive potential.

In this study, we demonstrated that plasma levels of miR-125b were upregulated in patients with EOC compared to healthy controls. Our results are consistent with an Indian study 52 and a Chinese study 53 which documented that circulating miR-125b levels were higher in women with EOC than in healthy controls.

However, Chinese and Italian studies performed on tissues 54-57 reported downregulation of miR-125b in EOC tissues compared with controls. This may be explained by the biological samples studied and by the different oncogenes and tumour suppressor genes targeted by miR-125b. The molecular mechanisms leading to the down or up-regulation of miR-125b in this type of cancer are not yet fully understood. To better validate our findings, we must study the expression of these miRNA in tissues from EOC patients and identify their target genes.

The present study found no significant difference in miR-21 and miR-125b levels when age, BMI, menarche, menopause, FIGO stage, disease recurrence, and distant metastases were considered. However, a large sample size and additional studies are needed to elucidate better the potential association between these clinicopathological features and dysregulated miRs.

In advanced EOC, the first line of chemotherapy consists of the combination of carbo/cisplatin and paclitaxel; unfortunately, 20% of patients do not respond to treatment 58. Our result indicates that decreased level of miR-125b is associated with chemoresistance. This is consistent with the findings of Chen and Sorrentino, who found downregulation of miR-125b in chemo resistant patients compared to those who responded to treatment 59,60. This can be explained in part by the fact that a significant decrease in miR-125b expression levels leads to an increase in the expression levels of the anti-apoptotic factor BCL-2, which is a direct target of this miR. In other studies, miR-125b was upregulated in chemo resistant patients 60,61, suggesting that the upregulation of miR-125b leads to significant inhibition of bak-1, a pro-apoptotic regulator involved in a variety of cellular activities and also a direct target of miR-125b. Consequently, downregulation of bak-1 suppresses cisplatin-induced cytotoxicity and apoptosis and, thus, resistance to cisplatin 61.

## Conclusion

The present study revealed significant upregulation of circulating miR-21 and miR-125b in EOC patients compared with healthy controls. Moreover, decreased miR-125b was detected in EOC patients who developed resistance to treatment, highlighting the potential role of miR-21 and miR-125b as future theranostic biomarkers in EOC.
